# Tracing the Evolution of Competence in *Haemophilus influenzae*

**DOI:** 10.1371/journal.pone.0005854

**Published:** 2009-06-10

**Authors:** Heather Maughan, Rosemary J. Redfield

**Affiliations:** Department of Zoology, University of British Columbia, Vancouver, British Columbia, Canada; St. Petersburg Pasteur Institute, Russian Federation

## Abstract

Natural competence is the genetically encoded ability of some bacteria to take up DNA from the environment. Although most of the incoming DNA is degraded, occasionally intact homologous fragments can recombine with the chromosome, displacing one resident strand. This potential to use DNA as a source of both nutrients and genetic novelty has important implications for the ecology and evolution of competent bacteria. However, it is not known how frequently competence changes during evolution, or whether non-competent strains can persist for long periods of time. We have previously studied competence in *H. influenzae* and found that both the amount of DNA taken up and the amount recombined varies extensively between different strains. In addition, several strains are unable to become competent, suggesting that competence has been lost at least once. To investigate how many times competence has increased or decreased during the divergence of these strains, we inferred the evolutionary relationships of strains using the largest datasets currently available. However, despite the use of three datasets and multiple inference methods, few nodes were resolved with high support, perhaps due to extensive mixing by recombination. Tracing the evolution of competence in those clades that were well supported identified changes in DNA uptake and/or transformation in most strains. The recency of these events suggests that competence has changed frequently during evolution but the poor support of basal relationships precludes the determination of whether non-competent strains can persist for long periods of time. In some strains, changes in transformation have occurred that cannot be due to changes in DNA uptake, suggesting that selection can act on transformation independent of DNA uptake.

## Introduction

Many bacteria develop a physiological state called natural competence, enabling cells to take up DNA from the environment and occasionally recombine it with the chromosome; a cell is said to be transformed if recombination changes its genotype. This genetically programmed process differs from artificially induced competence, where divalent cations or electric fields are used to permeabilize cell membranes. Competence has important implications for the ecology and evolution of bacteria: the nucleotides salvaged from the degradation of incoming DNA provide nutrients, and the alleles introduced by transformation can provide genetic novelty and templates for DNA repair.

Because the ability to be take up DNA and become transformed is sporadically distributed at all phylogenetic levels in bacteria, its evolutionary origin(s) are unclear. Many well-studied major clades have both transformable and non-transformable members, a state that is consistent with either a single origin and many losses or multiple independent origins (reviewed in [1], [2]. Comparison of the cellular machineries used for DNA uptake does not resolve the issue. Although almost all bacteria that have been studied use a modified type IV pilus system for DNA uptake, which would seem to support a single evolutionary origin [3], [4], the type IV pilus machinery also functions to generate forces for adhesion and motility [5]–[7] and may have been independently co-opted for DNA uptake in different lineages.

On a smaller scale but parallel to what is seen in the major clades, all species that have been examined include both transformable and non-transformable isolates [8]–[14]. This variability between strains may also account for the observed differences in competence at higher phylogenetic levels, as decisions about the competence of bacterial species are customarily based on the phenotype of a single strain. Differences between strains of the same species indicate that changes in competence are recent and provide a framework for identifying the microevolutionary changes responsible for the macroevolutionary patterns of competence.

*Haemophilus influenzae* is the model organism for studies of competence in the gamma-proteobacteria (e.g. [15]). Although *H. influenzae* is an obligate commensal of the human respiratory tract, it also causes opportunistic infections in both the respiratory tract and normally sterile sites, especially in infants, the elderly, and individuals with weak immune systems. In previous work we have characterized the variation in competence in *H. influenzae* by measuring the amount of DNA taken up and recombined by 35 genetically diverse strains [16]. Although most strains took up DNA and were transformed, the amounts of DNA taken up and the frequencies of transformation varied over several orders of magnitude. The goal of this paper is to place this variation into the context of the evolutionary relationships of these strains, to determine the extent to which loss and gain events have contributed to the present distribution of competence.

Previous work has been unable to establish the evolutionary relationships of *H. influenzae* strains; analysis using seven concatenated Multi-Locus Sequence Typing (MLST) loci resulted in tree topologies that did not provide strong support for most clades [17], [18]. The lack of resolution in the topologies from these studies may have had several causes: insufficient variation in the 3 kb of sequence used for the MLST analysis, the inability of parsimony reconstruction methods to accurately resolve the relationships of strains, and/or population processes that interfere with phylogenetic reconstruction, such as recombination between strains or changes in effective population size [19]–[21]. To infer the evolutionary relationships of the *H. influenzae* strains used in our studies of DNA uptake and transformation, we have now applied multiple inference methods to different data sets, including whole genome sequences.

## Results and Discussion

### Determining the evolutionary relationships of strains

This study used all of the strains whose competence we previously measured and whose seven MLST loci had been sequenced [16]. These strains had been chosen to include both close and distant relatives, according to the phylogeny in Meats et al. [18]. Most do not express a polysaccharide layer surrounding the cell (i.e., are nontypeable); however three serotype d and three serotype e strains were also included. For the present analysis we also included strain 3655 because its genome sequence is now available [22]. Strains and their properties are listed in Table 1.

**Table 1:** *H. influenzae* strains and their characteristics.

Strain^a^	Sero-type	Isolated from^b^	Uptake	TF	Instability (MLST)c,d	Instability (Gen)c,d	Instability (Comp)c,d
1247	nt	OM	8.3×10^−2^	1.7×10^−5^	13.80	n/a	n/a
PittDD	nt	OM	7.0×10^−1^	5.3×10^−3^	13.80	n/a	n/a
Rd*	d	N	1.6	1.0×10^−2^	13.80	1144.92	160.74
RM7033	d	P	1.7×10^−3^	5.9×10^−8^	13.80	n/a	n/a
RM7271	d	RI	5.4×10^−2^	1.2×10^−3^	13.80	n/a	n/a
RM7429	d	unk.	6.9×10^−2^	3.9×10^−3^	13.80	n/a	n/a
RM6181	e	B	2.7×10^−3^	5.5×10^−8^	16.30	n/a	n/a
1181	nt	OM	9.4×10^−3^	1.2×10^−6^	16.40	n/a	n/a
R2846*	nt	OM	1.7×10^−3^	1.7×10^−7^	17.10	1263.95	202.12
375	nt	OM	4.5×10^−1^	1.4×10^−2^	17.22	n/a	n/a
432	nt	OM	8.6×10^−3^	2.3×10^−7^	17.22	n/a	n/a
R2866*	nt	M	1.7×10^−1^	1.3×10^−4^	17.27	1176.09	178.06
22.4-21*	nt	HN	1.3×10^−3^	1.8×10^−7^	17.28	1255.80	177.39
1124	nt	OM	6.0×10^−2^	1.4×10^−3^	17.35	n/a	n/a
22.1-21*	nt	HN	6.6×10^−3^	2.2×10^−6^	17.42	1201.15	172.83
Eagan	b	M	5.4×10^−3^	5.4×10^−3^	17.63	n/a	n/a
PittHH*	nt	OM	3.0×10^−3^	2.8×10^−8^	17.81	1264.19	162.62
PittAA*	nt	OM	9.0×10^−3^	8.9×10^−3^	18.04	1266.07	157.07
1207	nt	OM	3.2×10^−2^	5.0×10^−4^	18.52	n/a	n/a
1209	nt	OM	8.1×10^−3^	6.0×10^−4^	18.52	n/a	n/a
1233	nt	OM	6.7×10^−3^	3.6×10^−4^	18.52	n/a	n/a
PittII*	nt	OM	5.6×10^−2^	2.7××10^−6^	18.62	1113.88	145.03
RM6169	e	A	1.1×10^−1^	7.2×10^−5^	18.67	n/a	n/a
PittEE*	nt	OM	3.1×10^−3^	2.1×10^−7^	19.60	1257.59	163.42
176	nt	OM	6.3×10^−3^	6.6×10^−7^	20.03	n/a	n/a
477	nt	OM	4.5×10^−2^	2.6×10^−8^	20.05	n/a	n/a
3655*	nt	OM	n/a	n/a	20.46	1214.35	166.26
1008	nt	OM	1.8×10^−2^	8.6×10^−6^	21.46	n/a	n/a
PittGG*	nt	O	1.0×10^−2^	2.7×10^−4^	21.46	1371.98	185.45
1158	nt	OM	5.4×10^−2^	1.2×10^−6^	22.00	n/a	n/a
RM6158	e	unk.	2.1×10^−3^	1.1×10^−8^	22.29	n/a	n/a
1159	nt	OM	1.0×10^−2^	4.9×10^−6^	22.38	n/a	n/a
PittBB	nt	OM	3.5×10^−3^	1.7×10^−8^	26.83	n/a	n/a
R3021*	nt	HN	3.7×10^−2^	2.1×10^−7^	30.47	1355.62	194.65
86028NP*	nt	OM	4.2×10^−3^	5.5×10^−6^	44.44	1321.30	185.74

Uptake data and transformation frequencies (TF) are from ref. [16]. Strains are sorted by their instability in the MLST dataset.

a Strains with genome sequence data available indicated by *.

b M: meningitis. N: nasopharynx; OM: otitis media; HN: Healthy nasopharynx; O: Otorrhea; P: Pneumonia; RI: Respiratory infection; B: Bronchitis; A: Asthma; Unk.: no information available.

c n/a: data not available. I = Instability.

**Figure 1:**
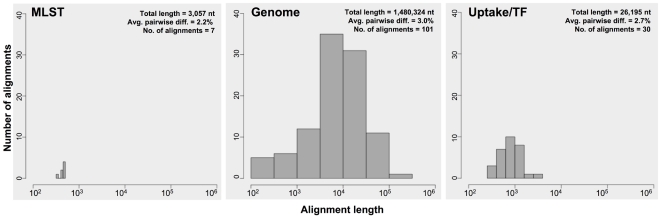
The distribution of alignment sizes is shown for each dataset used for phylogenetic analysis. Note the *x*-axis is a log scale.

Sequences from these strains were combined into three datasets: a Genome dataset, a MLST dataset, and an Uptake/transformation dataset. Genome dataset: Although strains differ at MLST loci by an average of 2.8% [18], not all of these sites are likely to be ‘informative’ for parsimony analysis. Therefore, we created a new dataset with greatly increased sequence data by aligning the genomes of the 13 strains for which genome sequencing has either been completed or is near completion, giving 1.5 Mb of aligned sequence (Figure 1). DNA uptake and transformation phenotypes have been characterized for 12 of these strains [16]. Sequences in this dataset differ by an average of 3.0%; because some genomes have not been completely assembled, it consists of 101 aligned blocks ranging in size from 150 to 108,151 nt (Figure 1). MLST dataset: To compare our results with previous studies, we also analyzed seven MLST loci from strains whose DNA uptake and transformation are known; strains in this dataset differed by an average of 2.2% (Figure 1). Uptake/transformation dataset: Reconstructing the evolutionary relationships of strains using only data from competence genes would allow us to compare the evolution of competence genes with evolution in housekeeping genes (MLST dataset) and the whole genome (genome dataset). Therefore, the third dataset consisted only of the 30 genes whose products are associated with DNA uptake or transformation (Table 2). This dataset contained the same strains as the Genome dataset; the sequences differed by an average of 2.7% (Figure 1).

**Table 2:** Genes in uptake/transformation dataset.

Gene name	Length (bp)	Predicted Function
*comA*	798	DNA uptake
*comB*	507	DNA uptake
*comC*	522	DNA uptake
*comD* $	414	DNA uptake
*comE*	1338	DNA uptake
*comE1*	339	DNA uptake
*comF*	687	DNA uptake
*comM* *	1530	Transformation
*comN*	513	DNA uptake
*comO*	717	DNA uptake
*comP*	684	DNA uptake
*comQ*	306	DNA uptake
*crp*	675	Regulation
*cyaA*	2532	Regulation
*dprA*	1122	Transformation
*icc*	825	Regulation
*ligA* %	558	Unknown
*murE*	1467	Unknown
*pilA*	450	DNA uptake
*pilB*	1395	DNA uptake
*pilC*	1221	DNA uptake
*pilD* #	693	DNA uptake
*pilF2*	540	DNA uptake
*radC*	705	Transformation
*rec-1*	1065	Transformation
*rec-2*	2367	DNA uptake
*ssb*	507	Transformation
*sxy*	654	Regulation
0365	1173	Unknown
0659	297	Unknown

Predicted functions related only to competence and transformation are listed.

$ frameshift in R3021.

* frameshift in strain R2866.

% nonsense mutation in PittAA.

# frameshift in strain 22.1-21.

**Figure 2:**
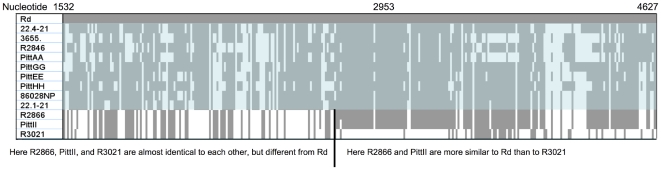
A putative recombination breakpoint identified using RDP. Only polymorphic sites are shown, corresponding to 3,095 bp of the original 7,014 bp in the aligned block. Grey squares indicate regions where other strains are identical to strain Rd and white squares indicate where strains are different. The black line indicates the approximate breakpoint. Strains without recombination in this region have been shaded.

Before undertaking phylogenetic analysis we first sought to determine the impact of recombination, which is predicted to shuffle the evolutionary histories of different genomic regions, resulting in conflicting phylogenetic signals [20]. However, recombination between strains does not always preclude the formation of well-defined lineages, as evidenced by the strong population structure in *Neisseria meningitidis* [23]. The majority of strains in our datasets were nontypeable; these are thought to have had more recombination than typable strains [18], [24], [25]. To determine whether our datasets contained recombinant regions we scanned each alignment block for signatures of recombination using several recombination detection methods. The first method used PhiTest to search for pairs of polymorphic sites whose evolutionary histories are incompatible [26]–[28]. The second method used the Recombination Detection Program (RDP) to run six different recombination detection methods, identifying regions as recombinant if all six methods agreed [29]. Because we obtained similar results with both methods we only discuss results from PhiTest. For the MLST dataset, two of the seven aligned blocks had significant evidence for recombination (*pgi* and *recA*; 29% of aligned blocks; Table 3). These results are consistent with another study that found high levels of recombination in *pgi* [25]. For the Genome dataset, 90% (91 of 101) of aligned blocks had significant evidence for recombination; because most of the exceptions were less than 1 kb long this suggests that recombination has been frequent throughout the genome (Table 3). In the uptake/transformation dataset, PhiTest identified recombination in 60% (18 of 30) of aligned blocks (Table 4). These results indicate that the MLST loci have less recombination between strains than the other two datasets; this may be because recombination in these housekeeping genes is deleterious. An example of a putative recombinant region is shown in Figure 2.

**Table 3:** Number of aligned blocks with evidence for recombination.

Dataset	PhiTest	RDP
MLST	2 of 7 aligned blocks	0 of 7 aligned blocks
Genome (>1000 nt)	88 of 90 aligned blocks	78 of 90 aligned blocks
Genome (<1000 nt)	2 of 11 aligned blocks	0 of 11 aligned blocks
Competence	18 of 30 aligned blocks	5 of 30 aligned blocks

**Table 4:** Clade with most frequent presence in topologies from each dataset (indicated by bold text).

Clade with highest support	MLST dataset	Genome dataset	Uptake/transformation dataset
1207 and PittHH	**5 of 7 topologies**	Strain 1207 not in dataset	Strain 1207 not in dataset
R2866 and PittII	1 of 7 topologies	**56 of 101 topologies**	11 of 30 topologies
PittAA and 3655	1 of 7 topologies	41 of 101 topologies	**14 of 30 topologies**

The support of each clade in other datasets is listed for comparison.

The next step in our analysis was to determine whether this recombination has diminished phylogenetic signal. We inferred individual MrBayes [30], [31] trees for each alignment block to see whether strain relationships varied between trees from different alignment blocks. When calculating a consensus tree from all trees in each dataset, we initially included only clades present in at least 80% of the trees produced from each dataset (7, 101 or 30); this produced star-like trees for all datasets. However the lack of resolution was not caused by this stringent cutoff, as a 60% cut-off gave essentially the same results, with only two clades supported by more than half of the trees (Table 4). Similar results were obtained when Parsimony methods were used for inference (data not shown).

**Figure 3:**
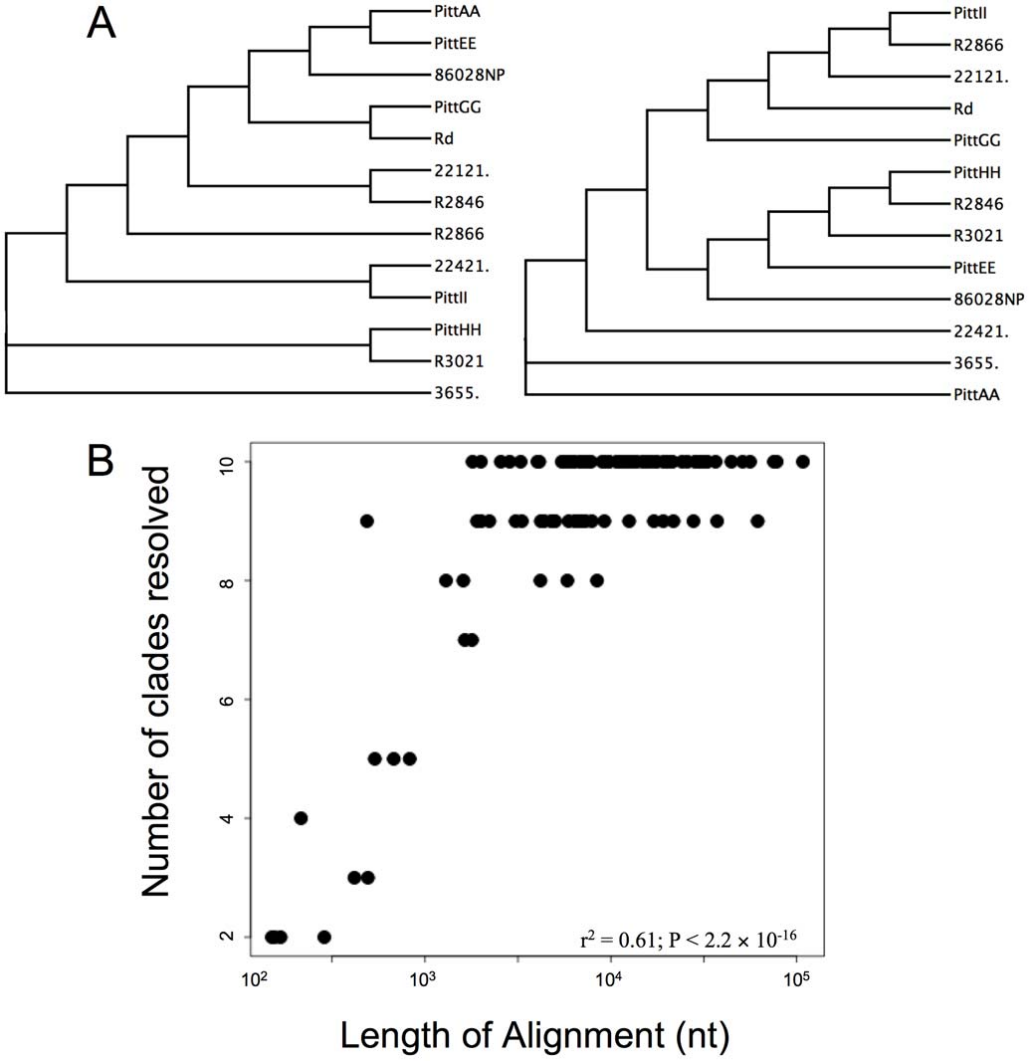
Factors resulting in the lack of resolution in consensus trees. (A) Two topologies from individual aligned blocks in the genome dataset. The two aligned blocks are of similar length (15,320 bp and 15,693 bp) but result in very different topologies. (B) The number of nodes resolved as a function of the aligned block length. Aligned block length explains a significant portion of the variation in the number of nodes resolved.

This lack of resolution could be due to disagreement between particular trees and/or to an insufficient number of informative sites in the aligned blocks. If the former, we would expect most trees to have at least some clades resolved, so we counted the number of clades resolved in each tree of the genome dataset (the other datasets had too few trees to be informative). 75% of trees had at least 9 of 11 possible clades resolved and 90% had at least 5 clades resolved, indicating that most trees had appreciable resolution despite the evidence for recombination within alignment blocks. Visual inspection of individual topologies confirmed this (see Figure 3A for an example). Thus disagreement between topologies resulted in the lack of resolution in the consensus tree, consistent with ubiquitous recombination throughout the genome.

Although individual trees disagreed in the clades they supported, many trees also had unresolved relationships. If this lack of resolution was caused by insufficient informative sites, the longer aligned blocks should have had more resolving power. Indeed, aligned block length explained a significant portion of the variation in the number of clades resolved (Figure 3B; r^2^ = 0.61; P<2.2×10^−16^). This relationship was partially driven by the 12 aligned blocks shorter than 1 kb; excluding these reduced the coefficient of determination but did not change it being significant (r^2^ = 0.21; P = 5×10^−6^). Therefore, the poor resolution of the MrBayes consensus trees is because some alignments had too few informative sites and because many of the clades supported in one tree were not supported in all trees.

Given the strong evidence for recombination, we proceeded with additional phylogenetic analysis using three inference methods specifically designed to deal with recombinant sequences: BUCKy [32], SplitsTree [27], [28], and ClonalFrame [33]. The results from each analysis are discussed in turn below.

**Figure 4:**
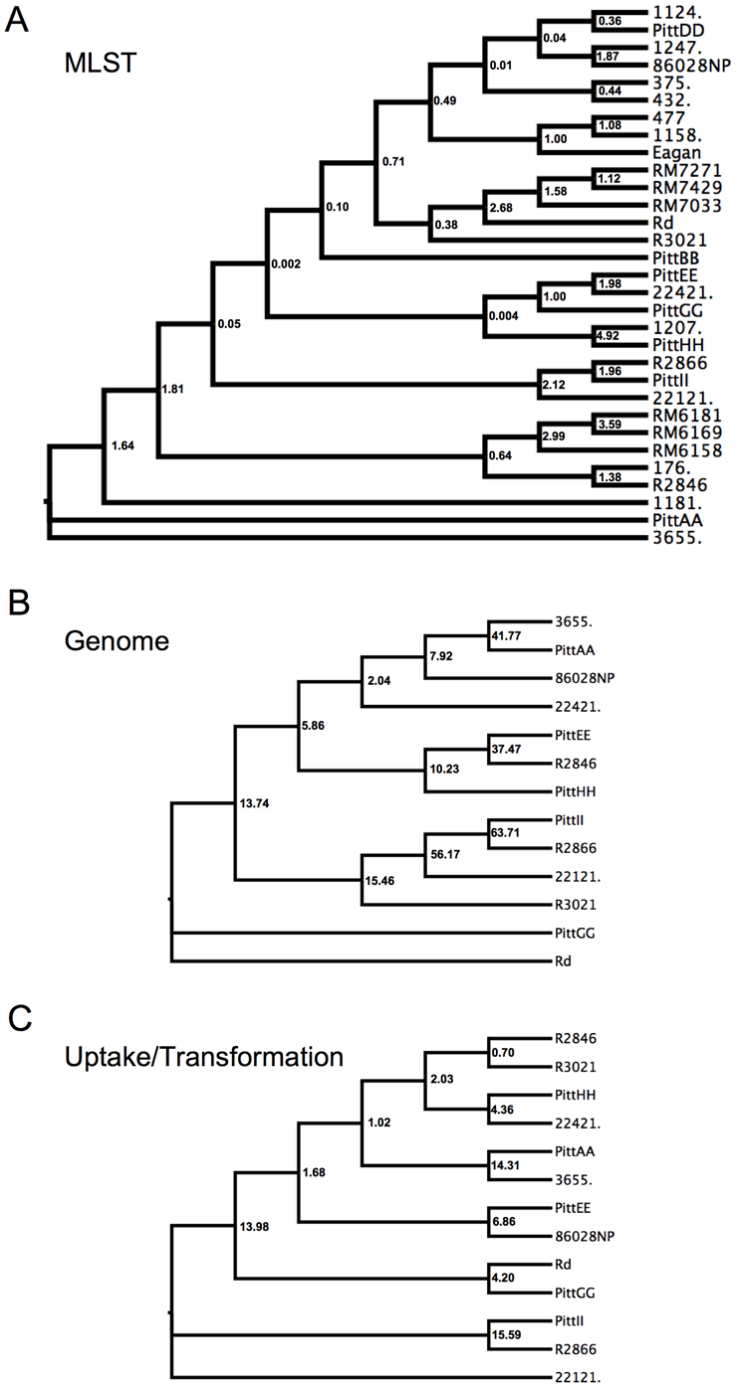
BUCKy trees for each of the three datasets. Numbers next to branching points indicate the concordance factors, which are indicators of the number of alignment blocks supporting the clade, out of 7, 101, and 30 for the MLST, Genome, and Uptake/Transformation dataset respectively.

BUCKy is a Bayesian method of inference that combines information from individual trees to infer one tree [32]; it differs from standard consensus tree approaches in considering the support for each clade in each input tree during inference of the final tree. For each of our three datasets, the distribution of trees from each alignment block (standard output of MrBayes) was used as input for BUCKy, giving one tree for each dataset (Figure 4). The numbers next to each clade in Figure 4 are concordance factors indicating the number of alignment blocks that have that clade. Few clades were supported by the majority of alignment blocks, indicating that combining information from multiple alignment blocks for inference in BUCKy does little to increase support for clades, especially those that are basal. Nevertheless some clades were found in trees from all datasets; these include the PittAA/3655 clade and the R2866/PittII/22.1-21 clade.

**Figure 5:**
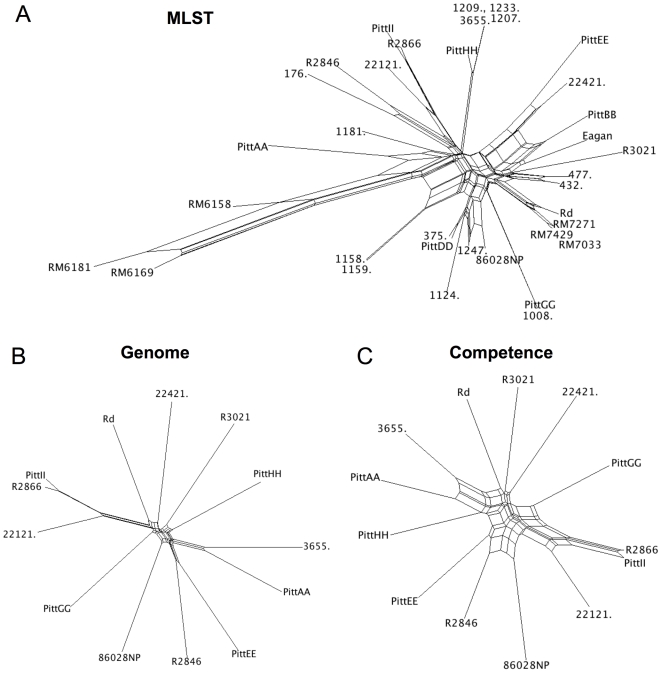
SplitsTree networks for each of the three datasets.

SplitsTree infers evolutionary relationships without assuming that evolution occurs only by bifurcation [27], [28], allowing the evolution of strains to be represented as a network containing both bifurcations and reticulations. We concatenated all alignment blocks for each dataset and used the resulting three alignments as input for SplitsTree. Consistent with the evidence for recombination given above, this showed extensive network structure, particularly in basal relationships (Figure 5). The R2866/PittII/22.1-21 clade was found in the network from each dataset; otherwise there was no agreement between the relationships inferred by BUCKy and SplitsTree for the Genome and Uptake/transformation datasets. In contrast, many of the clades in the MLST BUCKy tree were also found in the SplitsTree network, indicating that the MLST data may give more reproducible results with different inference methods.

**Figure 6:**
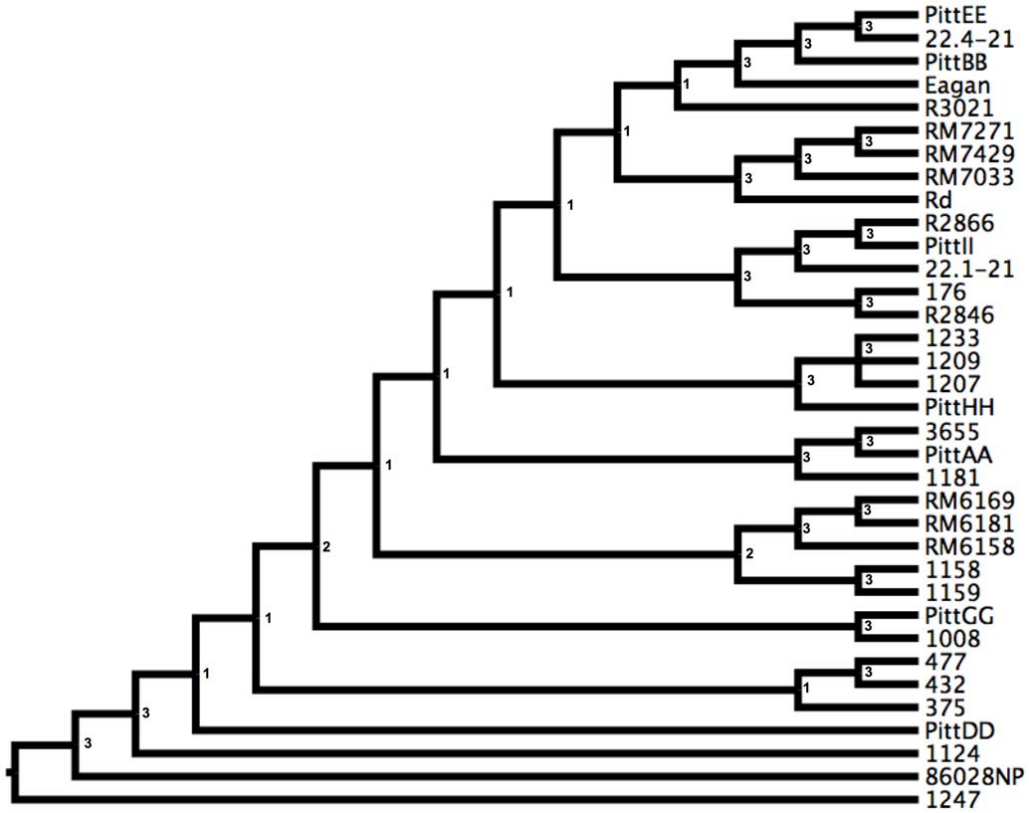
Extended majority rule consensus topology from three replicate ClonalFrame runs with the MLST dataset. The number next to each node indicates the number of replicate runs (out of three) that support that node.

Resolving strain relationships may improve if regions of recombination were removed from the alignment blocks. To do this, we used ClonalFrame, a coalescent-based Bayesian method that excludes putative recombinant regions before inferring strain relationships [33]. This method is computationally intensive so we only applied it to the MLST dataset. Three replicate runs were done to assess convergence of results, producing the consensus topology shown in Figure 6. Although most of the basal relationships in this tree differed from those in the BUCKy tree, many of the more recent relationships were in agreement.

Transformational recombination between strains may have been an important contributor to their recombinational histories. Because lab cultures of some strains exhibited much more recombination than others, we asked whether these strains' relationships were more difficult to resolve, using Mesquite to measure the stability of each strains' evolutionary relationships [34]. In this analysis strains that often have different close relatives in different trees are given high instability scores (see Table 1 for instability values). However highly transformable strains were not significantly less stable, as we found no significant relationship between these two properties in any of the three datasets (r^2^_MLST_ = 0.002, P = 0.8; r^2^_genome_ = 0.04, P = 0.53; r^2^_uptake/transformation_ = 0.007, P = 0.8). This lack of relationship between instability and transformation does not rule out a contribution of transformation to allelic exchange, but may simply indicate that the majority of recombination between strains occurs via conjugation and/or transduction.

### Tracing the evolution of competence

**Figure 7:**
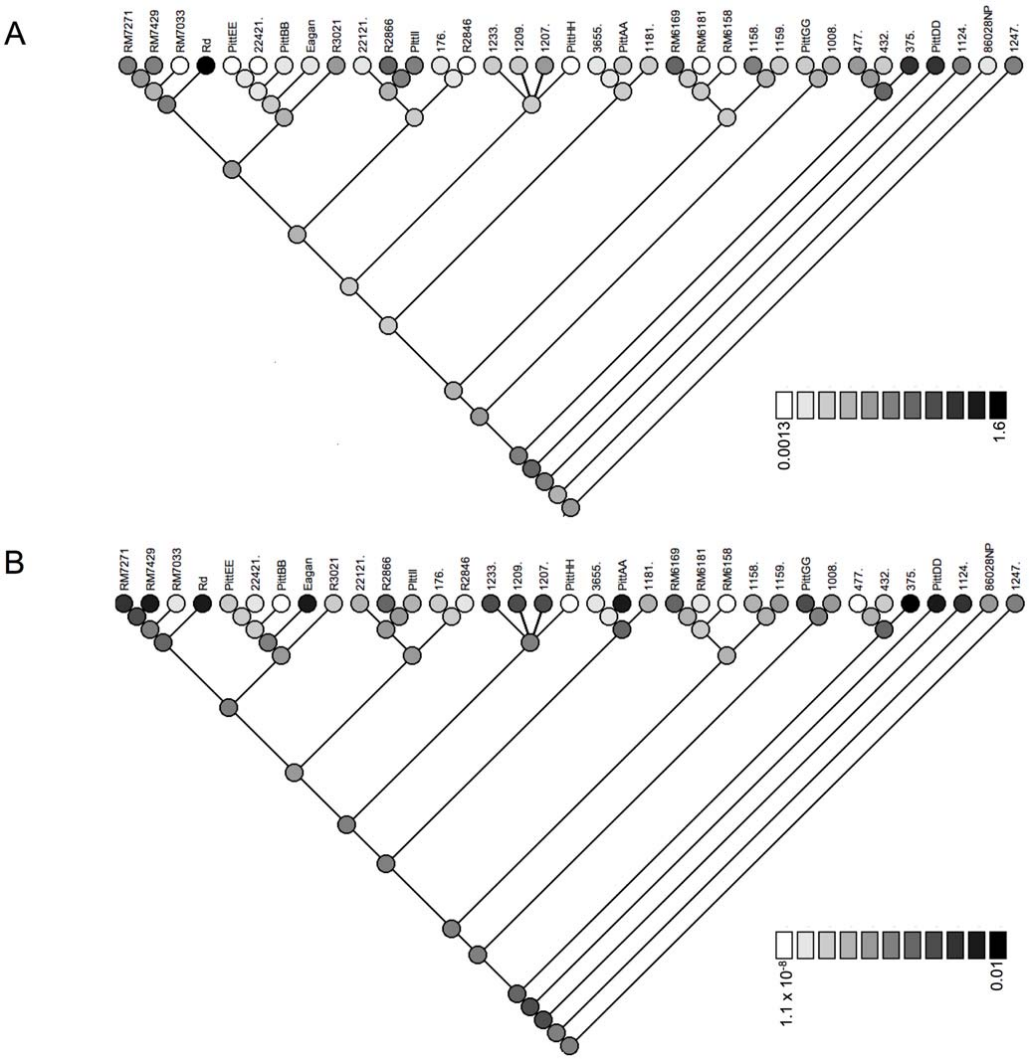
DNA uptake (A) and transformation frequencies (B) mapped onto the tree obtained from ClonalFrame. Light grey indicates low uptake (or transformation) and increasing intensity of grey indicates increasing uptake (or transformation). The numbers on the scale for DNA uptake and transformation correspond to the percent of radio-labeled DNA taken up per optical density unit and the transformation frequency, respectively [16].

The extensive variation we found in amounts of DNA taken up and recombined [16] must reflect changes in competence and transformability as lineages diverged from their common ancestor. We inferred the relationships of strains using multiple methods, but many of these relationships had low support or were not consistent between datasets or inference methods. Because the trees were inconsistent between different datasets and methods, we traced the evolution of DNA uptake and transformation separately on each of four trees: the MLST BUCKy tree (Figure 4A), the MLST ClonalFrame tree (Figure 6), the Genome BUCKy tree (Figure 4B), and the Uptake/transformation BUCKy tree (Figure 4C). Parsimony methods implemented in Mesquite were used to reconstruct ancestral states to infer whether DNA uptake and/or transformation have changed since each strain diverged from its most recent ancestor. For each strain, we compared the results between trees and discuss only increases or decreases that were consistent between all available trees. For each strain and for each tree, the change in DNA uptake and transformation since divergence from its ancestor is shown in Table 5. As visual examples of competence evolution, DNA uptake and transformation mapped onto the MLST ClonalFrame topology are shown in Figure 7.

**Table 5:** Change in DNA uptake and transformation during divergence of each strain from its most recent ancestor.

Strain	DNA uptake*				Transformation frequency			
	MLST BUCKy	MLST CF	Genome	Comp.	MLST BUCKy	MLST CF	Genome	Comp.
176	0.0025	0.0014	n/a	n/a	3×10^−7^	**−6×10^−8^**	n/a	n/a
375	0.3965	0.345	n/a	n/a	0.0141	0.014	n/a	n/a
432	**−0.0448**	**−0.0256**	n/a	n/a	**−6×10^−5^**	**−7×10^−7^**	n/a	n/a
477	0.0198	0.0105	n/a	n/a	**−1×10^−6^**	**−9×10^−7^**	n/a	n/a
1008	0.0073	0.001	n/a	n/a	**−1×10^−5^**	**−4×10^−5^**	n/a	n/a
1124	**−0.0689**	0.0029	n/a	n/a	5×10^−4^	0.0011	n/a	n/a
1158	0.03	0.0345	n/a	n/a	**−8×10^−7^**	**−1×10^−6^**	n/a	n/a
1159	**−0.0137**	**−0.0092**	n/a	n/a	3×10^−6^	3×10^−6^	n/a	n/a
1181	0.0008	**−0.0009**	n/a	n/a	**−8×10^−6^**	**−7×10^−6^**	n/a	n/a
1207	0.0239	0.0231	n/a	n/a	5×10^−4^	5×10^−4^	n/a	n/a
1209	**−0.0003**	**−0.0011**	n/a	n/a	6×10^−4^	6×10^−4^	n/a	n/a
1233	**−0.0017**	**−0.0025**	n/a	n/a	3×10^−4^	3×10^−4^	n/a	n/a
1247	0.0571	0.0407	n/a	n/a	**−4×10^−6^**	**−7×10^−6^**	n/a	n/a
3655	n/a	n/a	n/a	n/a	n/a	n/a	n/a	n/a
22.1-21	**−0.0086**	**−0.0089**	**−0.0124**	**−0.0118**	**−2×10^−6^**	**−2×10^−6^**	**−3×10^−6^**	**−6×10^−6^**
22.4-21	**−0.001**	**−0.0004**	**−0.0078**	**−0.0049**	**−4×10^−7^**	**−8×10^−8^**	**−5×10^−6^**	**−9×10^−7^**
86028NP	**−0.0219**	**−0.0174**	**−0.0043**	**−0.0035**	**−2×10^−5^**	**−3×10^−5^**	**−2×10^−6^**	2×10^−6^
Eagan	**−0.0091**	**−0.0027**	n/a	n/a	0.0053	0.0053	n/a	n/a
PittAA	n/a	n/a	n/a	n/a	n/a	n/a	n/a	n/a
PittBB	**−0.017**	**−0.0012**	n/a	n/a	**−5×10^−7^**	**−4×10^−7^**	n/a	n/a
PittDD	0.5707	0.5556	n/a	n/a	0.0044	0.0046	n/a	n/a
PittEE	**−0.001**	**−0.0004**	**−0.0015**	**−0.0048**	**−3×10^−7^**	**−5×10^−8^**	**−6×10^−7^**	**−3×10^−6^**
PittGG	**−0.0009**	**−0.0072**	**−0.0106**	**−0.0125**	2×10^−4^	2×10^−4^	2×10^−4^	2×10^−4^
PittHH	**−0.0055**	**−0.0062**	**−0.0045**	**−0.0032**	**−3×10^−5^**	**−4×10^−5^**	**−2×10^−6^**	**−1×10^−6^**
PittII	0.0036	0.0033	0.009	0.0131	**−9×10^−6^**	**−9×10^−6^**	**−8×10^−6^**	**−9×10^−6^**
R2846	**−0.0021**	**−0.0032**	**−0.0028**	**−0.0069**	**−2×10^−7^**	**−5×10^−7^**	**−6×10^−7^**	**−2×10^−7^**
R2866	0.1172	0.2228	0.1226	0.1267	1×10^−4^	1×10^−4^	1×10^−4^	1×10^−4^
R3021	**−0.0069**	0.0157	0.0218	0.0286	**−4×10^−6^**	**−4×10^−6^**	**−4×10^−6^**	**−2×10^−6^**
Rd	1.487	1.499	1.579	1.577	0.01	0.01	0.01	0.01
RM6158	**−0.0029**	**−0.005**	n/a	n/a	**−2×10^−7^**	**−3×10^−7^**	n/a	n/a
RM6169	0.0985	0.0971	n/a	n/a	7×10^−5^	7×10^−5^	n/a	n/a
RM6181	**−0.0087**	**−0.0101**	n/a	n/a	**−8×10^−7^**	**−1×10^−6^**	n/a	n/a
RM7033	**−0.0183**	**−0.0175**	n/a	n/a	**−1×10^−5^**	**−1×10^−5^**	n/a	n/a
RM7271	0.0117	0.0122	n/a	n/a	8×10^−4^	8×10^−4^	n/a	n/a
RM7429	0.0271	0.0277	n/a	n/a	0.0035	0.0035	n/a	n/a

Bold font indicates a decrease.

* The percent radio-labeled DNA taken up per optical density unit.

#### DNA uptake

The amount of DNA taken up by strains varied from 0.0013 to 1.6; these numbers indicate the percent of radio-labeled DNA taken up by cells, normalized by the optical density of culture [16]. Tracing the evolution of DNA uptake on each of the four trees showed that 28 of 35 strains took up different amounts of DNA than were predicted for their most recent ancestors. Thirteen of these changes were increases and fifteen were decreases. Increases ranged from 0.00075 to 1.58 in strains 1181 and Rd, respectively, and decreases ranged from 0.00035 to 0.07 in strains 1209 and 1124, respectively. Strains with the most pronounced changes in uptake all had increases; these were strains 375, PittDD, R2866, Rd, and RM6169.

#### Transformation

The transformation frequencies of strains varied from 1.1×10^−8^ to 0.01 [16]. Tracing changes in transformation frequency on each of the four trees indicated that all strains with a change in uptake had a change in transformation. Because DNA uptake is a prerequisite for transformation, we discuss these candidate changes in the context of the changes in DNA uptake inferred above. Eight strains with increased uptake had increased transformation whereas five strains with increased uptake had decreased transformation. Similarly, ten strains with decreased uptake had decreased transformation whereas five strains with decreased uptake had increased transformation. Changes in transformation that are in the same direction as changes in uptake are best interpreted as consequences of changes in DNA uptake. However, in ten strains uptake and transformation did not change in the same direction, suggesting that selective pressures may act independent of DNA uptake to increase or decrease the amount of incoming DNA that is recombined. One interpretation is that the costs and benefits of recombining DNA can be separate from the costs and benefits of taking it up. However, this could also be due to pleiotropic effects of selection for changes in recombinational repair or nuclease activity.

The sequence differences our previous study identified in some genes required for DNA uptake and transformation do not account for the phenotypic differences [16]. For example, strain 22.1-21's frameshift mutation in *pilD* inactivates a peptidase required for the assembly of uptake machinery in other bacteria, but this strain takes up significant amounts of DNA and routinely produces hundreds of transformants [4]. Equally perplexing is the *comM* frameshift in strain R2866. A *comM* mutant of strain Rd has normal DNA uptake and 300-fold reduced transformation [35], but this strain has higher uptake and transformation than its closest relatives. Pseudogene alleles were also identified in two other strains, R3021 (*comD*) and PittAA (*ligA*). However these genes' roles in uptake and transformation are unknown, and ancestral phenotypes could not be inferred because R3021 was placed differently in the different trees (Table 5) and PittAA's closest relative was strain 3665, whose uptake and transformation have not been measured. None of the other competence genes in these and other sequenced genomes had obvious defects, indicating that changes in competence phenotypes are due to single nucleotide polymorphisms.

What do these results tell us about the evolution of competence? Under the model proposed by Redfield et al. [36], ancestral *H. influenzae* was naturally competent but its descendants frequently lost competence by mutation. Although such losses might be transiently neutral or beneficial, the resulting lack of either nucleotides or recombination would be detrimental in the long term. Non-competent clades would then rarely persist for long periods of time, either because non-competent clades went extinct or because subsequent mutations restored competence. This model's prediction that most changes in competence should be recent events is consistent with the recent changes inferred above, but the poor support for basal clades and disagreement between the four trees makes it difficult to determine whether some strains have persisted despite ancestrally low competence. The strongest candidate is strain PittEE; although different trees assigned it different close relatives, it was always most closely related to a strain of low competence (strain 22.4-21 in the MLST trees, strain 86028NP in the Genome tree, and strain R2846 in the Uptake/transformation tree). Clades of lowly competent strains are not unique to *H. influenzae* as several clades of entirely non-transformable strains exist in *Pseudomonas stutzeri* [13].

The poor resolution of strain relationships in *H. influenzae* prevented identification of non-competent lineages that have persisted for longer periods of time, indicating that phylogenetic methods may not be well suited for tracing competence evolution in *H. influenzae*. As an alternative, the sequences of competence genes can provide evidence of the timing of loss of competence. For example, a strain with multiple inactivating mutations in several competence genes is likely to have lost competence long before a strain with a single inactivating mutation in one competence gene. None of the 13 sequenced genomes fall in the former category [16], so those that are not competent likely lost competence recently.

Because phenotypic variation can reveal differences in how selection has acted, understanding the molecular causes of competence differences may help shed light on the relative importance of its potential benefits. The best approach may be to compare the variation in competence with that in other bacterial phenotypes whose functions are undisputed, such as nutrient acquisition and DNA repair. Unfortunately, variation in even these straightforward phenotypes has not been rigorously documented. Although many pathways for nutrient acquisition appear to vary across different niches, consistent with well-established variation in resources [37], and many DNA repair pathways appear to be largely uniform across a wide range of niches, consistent with the ubiquity of endogenous damaging agents, we lack systematic data.

## Materials and Methods

### Datasets

#### MLST dataset

The aligned sequences of *adk*, *atpG*, *frdB*, *fucK*, *mdh*, *pgi*, and *recA* from all of the 35 strains listed in Table 1 were downloaded from http://haemophilus.mlst.net/.

#### Genome dataset

Genome sequences available for *H. influenzae* strains were downloaded from NCBI in Nov 2007. The genomes of strains 22.1-21, 22.4-21, PittAA, PittHH, PittII, R2846, R2866, and R3021 had not been completely assembled and so were only available as multiple contigs. Because no annotation was available we were unable to identify orthologs for these genomes.

Genomes were aligned using mauveAligner (Mauve version 2.1.1) [38], [39] with default parameters, which resulted in alignments of 116 genome segments. Ten segments were excluded from phylogenetic analyses after visual examination of each alignment because it was obvious that one or more sequences were not homologous. An additional 5 were excluded because they were short (<50 nt) and had no polymorphism. The lengths of the remaining 101 segments ranged from 150 to 108,151 nt, with a median of 7,901 nt and a mean of 14,700 nt. Gaps were removed from aligned blocks before phylogenetic inference in MrBayes (see below).

#### Uptake/transformation gene dataset

A list of *H. influenzae* Rd genes whose products regulate competence, contribute to DNA uptake machinery, or interact with incoming DNA has been previously compiled for the 13 strains with sequences available (Table 2; [40]). Briefly, competence gene sequences from strain Rd were used as queries in BLAST searches against the 13 strains with complete genome sequences available. Competence gene sequences were aligned manually or in CLUSTALW.

### Phylogenetic inference

#### MrBayes

The same parameters were used with each alignment block in MrBayes, except for the longest alignments, which required 2X–3X more generations to run. Most runs had 500,000 generations with printing every 100 generations. 0.25% generations were removed as burnin before consensus tree building. The General Time Reversible model with gamma distributed evolutionary rates, including a proportion of sites that are invariant, was chosen as the most appropriate model of nucleotide substitution in ModelTest [41].

#### BUCKy

For each dataset BUCKy [32]was run using default parameters and a burnin identical to that for MrBayes. The *a priori* level of discordance between input trees (α) was varied from its default value of 1 up to 10; this did not significantly change the results.

#### SplitsTree

Concatenated alignment blocks from each dataset were imported into SplitsTree [27], [28]. The NeighborNet method with uncorrected distances was used to create the networks shown in Figure 5. Network structure was insensitive to different models of nucleotide substitution and numbers of bootstrap replicates.

#### ClonalFrame

Three replicate runs were done in ClonalFrame [33]; these had 5×10^5^ iterations of burn-in and 5×10^6^ iterations after burn-in; output was recorded for the posterior sample at 1,000 iteration intervals. An 85% consensus topology from the three replicate runs was estimated in CONSENSE.

### Tests for recombination

Each aligned block was used as input for the PhiTest [26], as implemented in SplitsTree [27], [28] and the analyses were run using default parameters. RDP [42], Chimaera & MaxChi [43], [44], Bootscan [45], SiScan [46], and 3Seq [47] were also used in the Recombination Detection Program [29]; only those recombination events that were agreed upon by all six programs were retained.

### DNA uptake and transformation phenotypes

DNA uptake and transformation were measured as previously described [16]. Briefly, DNA uptake was measured by counting the amount of radio-labeled DNA retained by competent cells after 15 minutes of incubation. Raw measurements were corrected for cell number using optical density readings. Transformation was measured by providing competent cells with a cloned allele conferring resistance to the antibiotic novobiocin (nov). Transformation frequencies were obtained by dividing the number of nov resistant colonies (corrected for spontaneous *nov^R^* mutants) by the total number of colonies. Transformation frequencies were not dependent on divergence between donor and recipient alleles [16].

### Taxon instability

Taxon instabilities were calculated in Mesquite [34]. For each strain, the distance between it and all other strains was calculated for each tree pair, and the sum of all distances is the measure of instability.

### Mapping of DNA uptake and transformation phenotypes

DNA uptake and transformation values were mapped onto each of the four trees using Parsimony in the Trace Character History function in Mesquite [34]. Raw data values were log_10_-transformed before mapping to normalize their distribution.
